# A case report of Turner syndrome associated with fetal nuchal cystic hygroma and bilateral syndactyly of the hands and feet

**DOI:** 10.1186/s13052-019-0680-4

**Published:** 2019-07-18

**Authors:** Hai-Ying Chen, Jian-Qiong Zheng, Hong-Ping Zhang

**Affiliations:** Department of Obstetrics and Gynecology, Wenzhou People’s Hospital, Wenzhou Maternal and Child Health Care Hospital, The Third Clinical Institute Affiliated To Wenzhou Medical University, Wenzhou, 325000 China

**Keywords:** Turner syndrome, Nuchal cystic hygroma, Syndactyly

## Abstract

**Background:**

Turner syndrome (45,X), accounts for 1–2% of conceptions which typically miscarry early in the first trimester. Cases detected prenatally often present with cystic hygroma, which is an ultrasound marker for aneuploidy generally, but Turner syndrome particularly. In this study, we report a second trimester intrauterine fetal demise (IUFD), complicated by a marked cystic hygroma and bilateral syndactyly of the fingers and toes.

**Case presentation:**

A 25-year-old woman presented for her first prenatal visit at 22-week gestation with IUFD. Color Doppler ultrasound revealed a septated nuchal lymphatic hygroma and hydrops fetalis, characterized by edema of the whole body, substantial pleural effusion and abdominal fluid. Pregnancy was further complicated by oligohydramnios. Following labor induction, a stillborn female baby was delivered at 22 weeks gestation. Autopsy confirmed the presence of huge nuchal cystic hygroma (10 cm × 10 cm × 6 cm) and generalized edema. Bilateral, partial syndactyly involving digits 2–5 of the fingers and toes were also observed. Chromosomal analysis revealed a 45,X karyotype.

**Conclusions:**

We investigated an unusual case of severe septated nuchal cystic hygroma associated with bilateral syndactyly of the fingers and toes in a stillborn infant with Turner syndrome. Although cystic hygroma has been frequently reported in 45,X the severity is marked in this case. In addition, syndactyly is not a typical complication of Turner syndrome. This case emphasizes the importance of early ultrasound in pregnancy.

## Background

Redenbacher first described cystic hygroma in 1828 which is found in 1.0% of transvaginal ultrasound by first-trimester or early midtrimester [1]. Cystic hygroma is a macrocystic lymphatic malformation found in the posterior triangle between the neck and axilla in 75% of cases [2]. Cystic hygroma is regarded as an important marker for aneuploidy generally including trisomies but is a particularly common in Turner syndrome [3]. In fact the observation of cystic hygroma on ultrasound is attributable to Turner syndrome in 30–70% of cases [4]. Turner syndrome is one of the most common chromosomal disorders, thought to occur in 1–2% of all pregnancies, accounting for 10% of all spontaneously fetal loss and ~ 99% of all Turner syndrome conceptions are believed to miscarry [5]. The frequency of the condition in female newborns is approximately 1 in 2500 [6]. When diagnosed in the newborn period, Turner syndrome presents with puffiness of the hands and feet or redundant nuchal skin. They may also present with cardiac anomalies such as hypoplastic left heart or coarctation of the aorta. Most commonly, however, Turner syndrome presents later in childhood as short stature, or in adolescence when they fail to enter puberty [7].

In this study, we report an IUFD case of a fetus with Turner syndrome presenting at 22 weeks gestation with profound cystic hygroma and hydrops fetalis. **S**yndactyly of the fingers and toes was also observed. Written informed consent from the parents was obtained for this publication.

## Case presentation

**Fig. 1:**
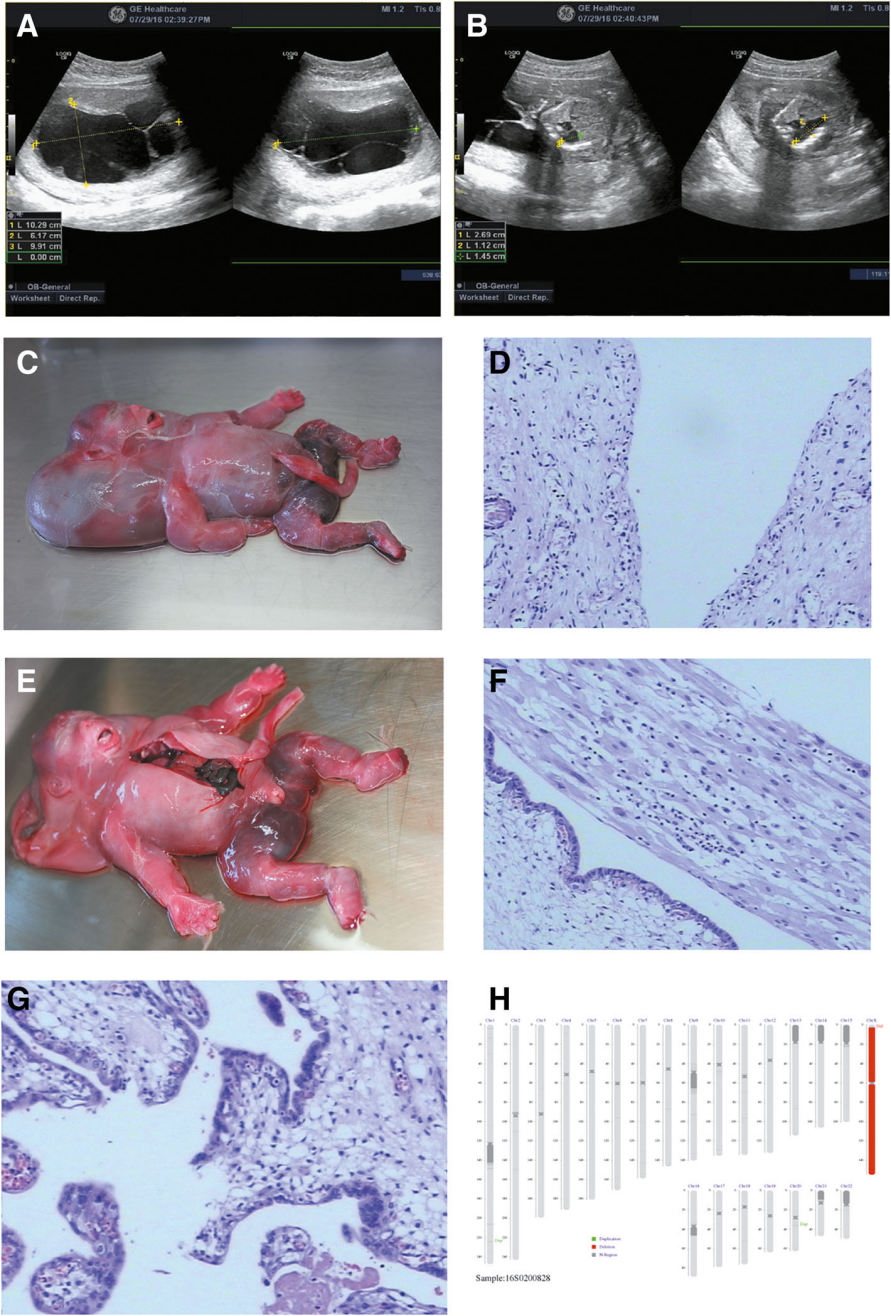
Clinical, histological and genetic observations. **a** Color Doppler ultrasound revealed nuchal lymphatic hygroma of about 62 × 99 × 103 mm as characterized by pathognomonic edema of the whole body. **b** Substantial pleural effusion and abdominal fluid of the fetus were also observed. **c**, **d** Autopsy data showed a huge nuchal cystic hygroma measured at 10 × 10 × 6 cm. **e** Webbed fingers and toes were also observed at autopsy. **f**, **g** pathological examination revealed severe placental chorioamnionitis. **h** Chromosomal karyotype analysis of the fetal buttock muscle tissue showed chromosome X deletion (45X)

A 25-year-old pregnant woman presented for her first prenatal appointment to the outpatient Department of Obstetrics at 22 week gestation. She was primigravida (G1P0) and reported an uneventful pregnancy prior to that point. She had not had any previous ultrasounds. Obstetric examination failed to detect a fetal heartbeat. Color Doppler ultrasound revealed nuchal lymphatic hygroma of about 62 × 99 × 103 mm and hydrops fetalis (Fig. 1a). Additionally, substantial pleural effusion and abdominal fluid of the fetus were also observed (Fig. 1b). Furthermore, the pregnancy was also complicated by oligohydramnios (AFV 11 mm). Upon inducing labor, a stillborn female baby was delivered at 22 weeks and 2 days of gestation, which weighed 600 g and the crown to heel measurement was 27 cm long (50th centile). Autopsy data showed a huge nuchal cystic hygroma measured at 10 × 10 × 6 cm (Fig. 1c, d), including 1000 ml of light red fluid, along with pathognomonic edema of the whole body. Partial, cutaneous syndactyly involving digits 2–5 of the fingers and toes were also observed on autopsy but, unfortunately, were not photographed clearly and radiographs were not performed. In addition, pathological examination revealed severe placental chorioamnionitis (Fig. 1f, g). Of note, no structural anomaly was found in the heart, lungs and kidneys. Chromosomal karyotype analysis of the fetal tissue showed monosomy X (45,X) (Fig. 1h).

## Discussion and conclusions

Cystic hygroma is a cystic lymphangioma frequently observed in soft tissues, such as neck (75%), axillae (20%), and retroperitoneum (5%) [2]. When detected on ultrasound, there is a high a priori probability of the fetus having Turner syndrome [4]. This was the case in the fetus reported who had a karyotype of 45,X. This case also presented with oligohydramnios and intrauterine fetal demise (IUFD), both of which are common complications of Turner syndrome [4, 8]. Turner syndrome is often associated with cardiac anomalies, renal deformity and low-set ears, none of which were detected in this case. This may reflect the variability of these features or the fact that a clinical geneticist did not examine the fetus. Of note, partial, cutaneous syndactyly of the fingers and toes has only rarely been reported in association with Turner syndrome, 45,X [9, 10], 45,X/46,Xr(X), 45,X/46,XY [11]. The syndactyly in this case may be caused by haploinsufficiency related to the 45,X karyotype though this is unlikely due to the rarity of the association. It is more plausibly caused by functional hemizygosity of a mutation on the remaining X chromosome. This is plausible given that isolated and syndromic syndactyly have been reported in association with X-chromosome genes. Alternatively it may be unrelated. Unexpectedly, serious placental infection, chorioamnionitis was detected by pathological examinations. However, it is unclear whether chorioamnionitis was associated with fundus disease and thus could have contributed to IUFD.

In this study, we report a case of Turner syndrome presenting with an unusually severe cystic hygroma and syndactyly of the fingers, toes. This case highlights the importance of a first trimester ultrasound which would have almost certainly have detected an increased nuchal translucency in this case and spared the mother the distress of an unexpected IUFD at 22 weeks gestation. It also adds another rare report of syndactyly in association with Turner syndrome. This study highlights the importance of ultrasound in late first trimester or early second trimester in recognizing unusual cases of severe aneuploidy presentations prior to IUFD late in the second trimester.

## Data Availability

All included.

## Abbreviation

IUFD: intrauterine fetal demise
